# Evaluation of S100A12 protein levels in children with familial Mediterranean fever

**DOI:** 10.3906/sag-2009-187

**Published:** 2021-06-28

**Authors:** Yelda TÜRKMENOĞLU, Elif GÜNEY, Diğdem BEZEN, Ahmet İRDEM, Biray ERTÜRK, Hasan DURSUN

**Affiliations:** 1 Department of Pediatrics, Okmeydanı Training and Medical Research Hospital, University of Health Science, İstanbul Turkey; 2 Department of Pediatric Endocrinology, Okmeydanı Training and Medical Research Hospital, University of Health Science, İstanbul Turkey; 3 Department of Pediatric Cardiology, Okmeydanı Training and Medical Research Hospital, University of Health Science, İstanbul Turkey; 4 Department of Medical Genetic, Okmeydanı Training and Medical Research Hospital, University of Health Science, İstanbul Turkey; 5 Department of Pediatric Nephrology, Okmeydanı Training and Medical Research Hospital, University of Health Science, İstanbul Turkey

**Keywords:** Children, familial Mediterranean fever, inflammation, S100A12

## Abstract

**Background/aim:**

Familial Mediterranean fever (FMF), the most common autoinflammatory disease in children, is characterized by recurrent febrile episodes. FMF is known to progress with chronic inflammation, particularly during attack periods. This study aimed to investigate the relationship of S100A12, an inflammatory marker, with attacks and inflammatory events in FMF patients.

**Materials and methods:**

The study included 57 patients diagnosed with FMF, 43 in an attack-free period and 14 in an attack period, and 31 healthy children as the control group. Only white blood cell (WBC) count, C-reactive protein (CRP) level, erythrocyte sedimentation rate (ESR), and S100A12 level were analyzed in the control group. In addition, serum amyloid A (SAA), and fibrinogen levels were measured, and a mutation analysis was performed in the patient group. The results were compared among the attack-free period, acute attack FMF and control groups.

**Results:**

The mean age of patients and control group was 10 (2.5–18) and 9.5 (2.5–16) years, respectively. The CRP (p = 0.001), S100A12 (p = 0.003) and ESR (p= 0.001) values differed significantly between the FMF and control groups. S100A12 level (p = 0.027), WBC count (p = 0.003), CRP level (p = 0.0001), ESR (p = 0.004), and fibrinogen level (p = 0.001) differed significantly between the acute attack and attack-free period groups. SAA level (p = 0.05), ESR (p = 0.001), fibrinogen level (p = 0.001), WBC count (p = 0.001), and S100A12 level (p = 0.027) were higher in M694V homozygous FMF patients than in other FMF patients.

**Conclusion:**

Patients with FMF had higher S100A12 levels than the control group, while the mean S100A12 concentration was higher in acute attack period patients than in attack-free period patients. S100A12 level might be an important indicator in the monitoring of chronic inflammation in patients with FMF.

## 1. Introduction

Familial Mediterranean fever (FMF) is a self-limited, autosomal-recessive autoinflammatory disease, characterized by recurrent febrile attacks and sterile polyserositis [1–4]. The Mediterranean Fever (MEFV) gene, which is localized on the chromosome 16p13.3, causes FMF, which is prevalent in the Eastern Mediterranean region [5]. Various MEFV mutations are known to cause FMF (M694V, E148Q, M694I, V726A, and M680I), but the most severe is reportedly a homozygous M694V mutation [2–4,6]. Defects in the MEFV gene lead to a pro-inflammatory response via interleukin-β by disrupting the synthesis of pyrin (marenostrin), which regulates neutrophil activity [7,8].

FMF is a disease that typically achieves clinical quiescence between irregular acute febrile episodes. There may be episodes with and without acute attacks in the course of FMF. Serum amyloid A (SAA), an acute-phase reactant produced in response to infection, inflammation, and trauma, is primarily synthesized in the liver. During the acute febrile attacks, an increase in biologically acute phase reactants such as white blood cell (WBC) count, C-reactive protein (CRP), erythrocyte sedimentation rate (ESR), and SAA can be observed. Diagnosing FMF becomes difficult due to the absence of a specific laboratory test, and findings that mimic infections, malignancies, and other autoimmune diseases [2–4,6]. The diagnosis and treatment are delayed due to an increase in similar inflammatory laboratory parameters (WBC count, CRP, ESR, and SAA); hence, there is a need for other diagnostic tests.

S100 proteins is a group of proinflammatory mediators released from human neutrophils that belong to a Ca+-binding protein family. The S100 proteins exhibit cytokine-like extracellular functions. The phagocyte-specific S100 proteins are known as A8, A9, and A12 (calgranulin C) and are released from monocytes and granulocytes. S100A12 is a member of the damage-associated molecular pattern molecule family and these proteins, after being released from activated or necrotic cells, induce an inflammatory response and the accumulation of immune cells in the damaged tissue [9]. The S100A12 gene is approximately 4.1 kilobase pairs (kbp) long and consists of three exons, which are divided by 900 and 400 bp introns. The first exon is untranslated and comprises 48 nucleotides. A classical TATA box (TATAAA) is located 30 nucleotides upstream of the transcription initiation site. The protein is encoded by sequences in exons 2 (138 nucleotides) and 3 (138 nucleotides). S100A12 is generally expressed by granulocytes and acts on the receptor for advanced glycation end products. The activation of this receptor induces the proinflammatory response via nuclear factor-kappa B in leukocytes and endothelial cells, and this protein is reportedly a marker for granulocyte activation that is more useful than CRP and ESR for detecting inflammatory activity. The amount of S100A12 protein is also known to increase in cases of malignancies, obstructive sleep apnea, autoimmune diseases, systemic lupus erythematosus, juvenile arthritis, and FMF [10–14].

The primary purpose of this study was to determine the relationship between S100A12 and chronic inflammation in children with FMF. The secondary purpose was to determine the association between S100A12 protein and other inflammation markers during the attack and attack-free periods as well as the association between this protein and other inflammatory markers in the attack period with the mutation findings.

## 2. Materials and methods

### 2.1. Study design

A- case control study with a nested cross-sectional assessment of acute attack period effects was performed at the Okmeydanı Training and Research Hospital between February 1, 2019 and July 1, 2019. Written approval was obtained from the local ethics committee of our hospital prior to the study, which was performed in accordance with the principles of the World Medical Association Declaration of Helsinki. The participants or their families were informed about the aims and scope of the research. Written informed consent was obtained from patients and their parents if the patient was older than 9 years or only from the parents if the patient was younger.

### 2.2. Patient populations

The study group initially consisted of 64 children with FMF aged 2.5–18 years who were followed up in our hospital’s pediatric nephrology outpatient clinic. We included healthy 36 children, who applied to the Pediatric Outpatient Clinic of our hospital for routine examination and had no history of fever, infection, malignancy, known chronic disease or drug use, as the control group. Seven patients and 5 healthy children with for whom patient records or laboratory tests were missing were excluded from the study. Overall, the study sample consisted of 88 individuals: (57 patients and 31 healthy controls). 

The Tel-Hashomer criteria were used to diagnose FMF [2–4,6,15]. Patients taking anti-inflammatory medications and anticoagulants, and those over 18 years or under 2.5 years old were excluded from the study. FMF patients were divided into two groups: attack-free patients and those in the attack period. Patients with joint, chest, or abdominal pain of unknown origin on the day of sample collection were considered attack FMF patients regardless of their laboratory values. Detailed stories of all patients were recorded, and the body mass indexes were calculated. Colchicine dosage and other treatments received by the patients were noted. In addition, the genetic analysis results of the FMF patients were examined. 

### 2.3. Laboratory tests

Blood samples were drawn to determine WBC count, CRP levels, ESR, and S100A12 level of individuals in the control group. In addition, SAA and fibrinogen levels were determined and mutation analyses were performed in the FMF group.

Genetic analysis:

Peripheral blood samples were used for DNA isolation. Two milliliters of peripheral blood was collected into an EDTA tube for DNA isolation. The DNA obtained was amplified by polymerase chain reaction (PCR) using primers specific for exon 2 (forward (F): 5′-GAGCAAACGCAGAGAGAAGG-3′ and reverse (R): 5′-TAGGTCGCATCTTTCCCGAG-3′), exon 3 (F: 5′-GTCCAGCTGCTCTTCTGTGA-3′, R: 5′-CTGCCTTCTCCTCCCCATAG-3′), exon 5 (F: 5′-ATGGTTGGGCAGATCAGGAA-3′, R: 5′-CACTTTTCAGGGACAGGCAC-3′) and exon 10(F:5′-AAATGAGTACCAGGCGTCCA-3′, and R:5′-AGGAGCTGTGTTCTTCCCTC-3′) sequences of the MEFV gene (Gene ID: 4210). The resulting fragments were sequenced using next generation sequencing for mutation analysis.

For the S100A12 measurement, Elabscience Human ELISA kits (Shanghai, China, 2019) were used. For the S100A12 test, 5 mL blood samples were taken from FMF patients and healthy controls, centrifuged in a 4000-RPM centrifuge for 10 min to obtain serum samples and stored at (–80) ºC until the study. All frozen samples were thawed at room temperature for the S100A12 test, and the ELISA kits were used to quantify serum human S100A12 protein concentrations and the results were recorded in nanograms per milliliter.

### 2.4. Statistical analyses

The statistical analysis was performed using SPSS software (v:17.0; SPSS Inc., Chicago, IL, USA). If the distribution of the continuous variables was normal, they were presented as mean ± SD (p > 0.05 on the Kolmogorov–Smirnov or Shapiro–Wilk test; (n < 30)). If the continuous variables were not normally distributed, they were described as the median (min-max). Intergroup comparisons were analyzed using Student’s t-test variance for normally distributed data, while the Mann–Whitney U test were used to examine non-normally distributed data. Receiver operating characteristic (ROC) curves were constructed and the areas under curve (AUC) and the sensitivity (sen.), and the specificity (spe.) were calculated. Spearman correlation test was used to examine the relationship between variables in the groups p-values < 0.05, were considered statistically significant. 

## 3. Results

The mean FMF patient age was 10 (2.5–18) years, while that of the controls was 9.5 (2.5–16) years. There was no statistically significant intergroup difference in age (p = 0.808). The clinical findings of the 57 FMF patients included in the study are shown in Table 1. The patient group consisted of 31 (54.4%) boys, whereas the control group consisted of 13 (41.9%) boys. There was no statistically significant intergroup difference in sex (p = 0.372). The four most common clinical findings in the patient group were abdominal pain (96.5%), fever (89.6%), leg pain (56.1%), and joint pain (54.4%). One of the patients underwent an appendectomy.

**Table 1:** Patients’ clinical symptoms at the time of admission.

Clinical characteristics	Patients (n)	%
Male/female	31/26	54.4/45.6
Abdominal pain	55	96.5
Fever	54	94.7
Exertional leg pain	32	56.1
Arthritis/arthralgia	31	54.4
Myalgia	18	31.5
Erysipelas like erythema	8	14.0
Splenomegaly	5	8.8
Peritonitis	3	5.3
Pleuritis	2	3.5
Family history	30	52.6

The patients’ and controls’ demographic and anthropometric characteristics and laboratory findings are shown in Table 2. There were no significant intergroup differences in mean age, weight, height, body mass index, hemoglobin, hematocrit, or WBC or platelet counts. There were statistically significant differences in CRP level (p = 0.001), S100A12 level (p = 0.003), and ESR (p = 0.0001) between the FMF and control groups.

**Table 2:** Patients’ and controls’ demographic and laboratory data.

Parameters	Patients (n = 57)Median (min-max)	Controls (n = 31)Median (min-max)	p
Age (years)	10 (2.5–18)	9.5 (2.5–16)	0.808
Weight (kg)	31 (13–68)	32 (13–73)	0.938
Height (cm)	135 (86–170)	135 (89–170)	0.985
WBC count (103/mm3)	8.2 (4.0–18.5)	7.26 (4.8–11.8)	0.129
CRP (mg/L)	3.6 (0.13–157.5)	0.4 (0.2–3.7)	0.001*
S100A12 (ng/mL)	435.2 (30–1644)	224.3 (20–897)	0.003*
ESR (mm/h)	26.49 (3–116)	10.74 (6–15)	0.0001*
	Mean ± SD	Mean ± SD	
BMI (kg/m2)	18.3 ± 3.3	17.8 ± 3.5	0.539
Hemoglobin (g/dL)	12.4 ± 1.4	12.6 ± 1.1	0.396
Hematocrite (%)	37.2 ± 3.5	37.1 ± 3.0	0.930
Platelet count (103/mm3)	298.9 ± 68.0	294.8 ± 51.9	0.772

*p < 0.05; Student t or Mann–Whitney U tests, CRP: C-reactive protein, ESR: erythrocyte sedimentation rate, BMI: body mass index, CRP: C-reactive protein, SD: standard deviation; WBC: white blood cell.

Patients were divided into attack-free period and acute attack FMF groups. There were statistically significant differences in the S100A12 (p = 0.027) and CRP (p = 0.001) levels between the acute attack and attack-free period patients. The differences in WBC count (p = 0.003), SAA level (p = 0.0001), ESR (p = 0.004), and fibrinogen level (p = 0.001) were also statistically significant between the attack-free period and acute attack groups (Table 3).

**Table 3:** Demographic and laboratory findings of acute attack period versus attack-free period groups.

Parameters	Attack free period (n = 43)Median (min-max)	Acute attack period (n = 14)Median (min-max)	p
Age (years)	10 (2.5–19)	9.7 (3.5–16)	0.662
Weight (kg)	30 (13–68)	36 (17–63)	0.738
Height (cm)	134 (86–165)	135 (103–170)	0.704
WBC count (103/mm3)	7.26 (4.8–15.8)	11.7 (4.9–18.5)	0.003*
CRP (mg/L)	1.6 (0.1–15.7)	34.9 (0.4–131.9)	0.0001*
S100A12 (ng/mL)	370 (30–1644)	634 (245–1460)	0.027*
ESR (mm/h)	14 (6–116)	40 (3–94)	0.004*
Serum amyloid A (mg/dL)	0.6 (0.1–62.0)	32.0 (0.09–138)	0.0001*
	Mean ± SD	Mean ± SD	
BMI (kg/m2)	18.2 ± 3.3	18,1 ± 3.3	0.650
Hemoglobin (g/dL)	12.3 ± 1.2	12.4 ± 1.9	0.774
Hematocrit (%)	36.9 ± 2.9	37.7 ± 5.0	0.853
Platelet count (10 3/mm3)	303.1 ± 72.2	286.0 ± 53.6	0.528
Fibrinogen (g/L)	2.6 ± 0.6	3.4 ± 0.8	0.001*

*p < 0.05; Student t or Mann–Whitney U tests, CRP: C-reactive protein, ESR: erythrocyte sedimentation rate, BMI: body mass index, CRP: C-reactive protein, SD: standard deviation; WBC: white blood cell.

The attack-free period and control groups were also compared in terms of S100A12 and other acute-phase reactants (Table 4). Statistically significant differences were found in CRP level (p = 0.001), S100A12 level (p = 0.030), and ESR (p = 0.002) between the attack-free and control groups. On the other hand, WBC count and fibrinogen levels did not differ significantly between the attack- free period and control groups (p > 0.05).

**Table 4:** Demographic and laboratory findings of attack-free period versus healthy controls.

Parameters	Attack free period (n = 43)Median (min-max)	Healthy controls (n = 31)Median (min-max)	p
Age (years)	10.0 (2.5–19.0)	9.5 (2.5–16.0)	0.969
Weight (kg)	30.0 (13.0–68.0)	32.0 (13.0–73.0)	0.952
Height (cm)	134.0 (86.0–165.0)	135.0 (89.0–170.0)	0.902
WBC count (103/mm3)	7.26 (4.8–15.8)	7.26 (4.8–11.8)	0.446
CRP (mg/L)	1.6 (0.1–15.7)	0.4 (0.2–3.7)	0.001*
S100A12 (ng/mL)	370 (30–1644)	224.3 (20–897)	0.030*
ESR (mm/h)	14 (6–116)	10.74 (6–15)	0.002*
Parameters	Mean ± SD	Mean ± SD	
Hemoglobin (g/dL)	12.3 ± 1.2	12.6 ± 1.1	0.344
Hematocrit (%)	36.9 ± 2.9	37.1 ± 3.0	0.873
Platelet count (103/mm3)	303.1 ± 72.2	294.8 ± 51.9	0.498

*p < 0.05; Student t or Mann–Whitney U tests, CRP: C-reactive protein, ESR: erythrocyte sedimentation rate, CRP: C-reactive protein, SD: standard deviation; WBC: white blood cell.

Correlation tests were performed to investigate the relationship between S100A12 level and other acute-phase reactants in the attack-free period group, acute attack group, and all FMF patients (Table 5). There was a positive correlation between S100A12 and CRP levels (p = 0.002), between S100A12 level and ESR (p = 0.001), and between S100A12 and fibrinogen levels (p = 0.009) in all patients. There was a positive correlation between S100A12 level and ESR (p = 0.043) in the attack-free period group, while there was no correlation between S100A12 level and other acute-phase reactants in the attack group. 

**Table 5:** Correlation between S100A12 level and other acute-phase reactants in the attack-free period, acute attack period, and all patients.

Parameters	S100A12 (ng/mL)					
	All patients(n = 57)		Attack-free periodpatients (n = 43)		Acute attackperiod (n = 14)	
	r	p	r	p	r	p
WBC count (103/mm3)	0.195	0.146	0.044	0.788	-0.037	0.901
CRP (mg/L)	0.409	0.002*	0.092	0.559	0.313	0.275
ESR (mm/h)	0.430	0.001*	0.310	0.043*	0.476	0.085
SAA (mg/dL)	0.172	0.200	-0.027	0.866	0.018	0.951
Fibrinogen (mg/dL)	0.341	0.009*	0.115	0.463	0.382	0.177

Spearman correlation analysis, *p < 0.05, APR: acute phase reactants, WBC: white blood cell, CRP: C reactive protein, ESR: erythrocyte sedimentation rate, SAA: serum amyloid A.

The receiver operating characteristic (ROC) curve was also plotted for S100A12 and other acute-phase reactants (Table 6, Figure). To determine the cut-off value for S100A12, the area under the ROC curve (AUC) was 0.62 (95% confidence interval, 0.42–0.82). Similarly, AUC, cut-off value, sensitivity, and specificity values were determined for CRP, ESR, SAA, fibrinogen, and WBC.

**Table 6:** Receiver operating characteristic curve analysis of acute-phase reactants in Familial Mediterranean Fever patients.

Test result variable (s)	p	AUC(Area under curve)	AUC 95% Confidence interval		Cut off value	Sen. %
			Lower bound	Upper bound		
CRP (mg/L)	0.0001	0.832	0.69	0.97	4.80	85.7
ESR (mm/h)	0.004	0.758	0.58	0.94	26.5	78.6
S100A12 (ng/mL)	0.049	0.620	0.42	0.82	367	64.3
SAA (mg/dL)	0.0001	0.825	0.65	1.00	8.00	85.7
Fibrinogen (mg/dL)	0.002	0.777	0.62	0.93	2.96	71.4
WBC count (103/mm3)	0.003	0.766	0.58	0.95	8.76	78.6

AUC: Area under curve, CRP: C reactive protein, ESR: ethrytrocyte sedimentation Rate; FMF: Familial Mediterranean fever; SAA: Serum amyloid A, Sen: sensitivity; Spe: specifity; WBC: white blood cell.

**Figure:**
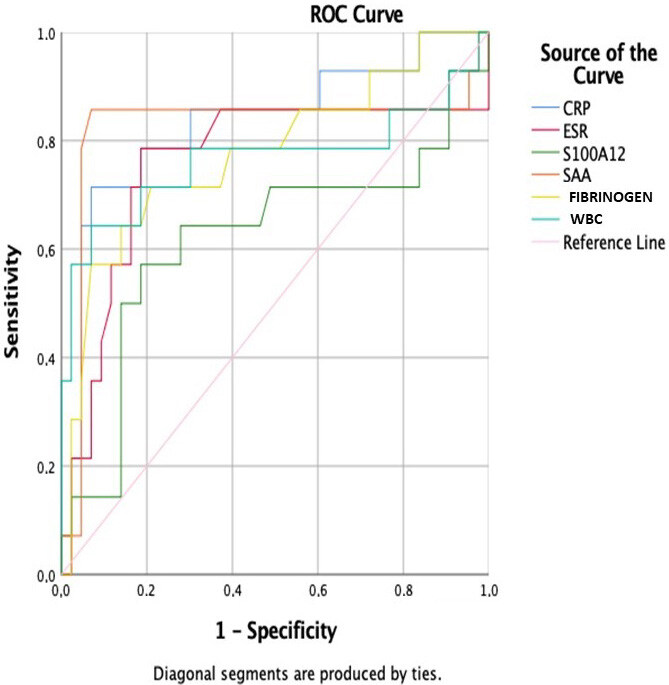
ROC curve of C reactive protein, erythrocyte sedimentation rate, S100A12 protein, serum amyloid A, fibrinogen, white blood cell count in FMF patients.

The genetic results of the FMF patients are shown in Table 7. The most common mutations in our patients were the M694V heterozygous mutation (in 26 patients) and R202Q heterozygous polymorphism. While an M694V mutation was seen in 35 total patients, an R202Q polymorphism in the exon 2 MEFV gene region was detected in 31 patients, 26 of whom (74.2%) had an M694V mutation. The mutation analysis was negative in one of the acute attack period FMF patient, while M694V heterozygous and other mutations were seen in 5 patients. There was no statistically significant difference in S100A12 between patients with or without the M694V mutation (p = 0.866).

**Table 7:** Genetic results of familial Mediterranean fever patients.

Gene mutations	Patients	
	n	(%)
M694V/M694V	5	8.77
M694V/M694V and R202Q/R202Q	5	8.77
M694V/N and R202Q/R202Q	3	5.26
M694V/N and R202Q/N	16	28.07
M694V/M680I and R202Q/N	2	3.5
M694V/E167A	1	1.75
M694V/N	3	5.26
R202Q/N	5	8.76
E148Q/E148Q	1	1.75
E148Q/N	6	10.52
M680I/N	2	3.5
E726A/RS61752717	2	3.5
E167D/E148V	1	1.75
RS224205/RS2894057	1	1.75
M694I/R761H	1	1.75
F479L/E167A	1	1.75
No mutation	2	3.5
Total	57	100

Patients with homozygous gene mutation for M694V and all patients with other homozygous, heterozygous, and compound heterozygous gene mutations were compared in terms of S100A12 and other parameters (Table 8). S100A12 levels were significantly higher in M694V homozygous patients than the patients with other mutations (p = 0.027). In addition, ESR (p = 0.001), fibrinogen level (p = 0.001), absolute neutrophil count (p = 0.004), and WBC count (p = 0.004) were significantly higher in the M694V homozygous group than the patients with other mutations.

**Table 8:** Comparison of acute-phase reactants of patients with M694V homozygous mutation versus other mutations.

Parameters	M694V Homozygous(n = 10) Mean ± SD orMedian (min-max)	Other mutations(n = 45) Mean ± SD orMedian (min-max)	p
S100A12 (ng/mL)	635 (245–1460)	370 (30–1644)	0.027*
SAA (mg/dL)	25.24 (0.64–57.80)	8.91 (0.06–138.00)	0.050
ESR (mm/h)	51.50 (15.00–116.00)	21.17 (3.00–62.00)	0.001*
CRP (mg/L)	33.06 (1.68–102.38)	13.39 (0.13–157.47)	0.081
Fibrinogen (g/L)	3.55 ± 1.03	2.65 ± 0.59	0.001*
ANC (103/mm3)	7191.00 ± 4112.59	4401.70 ± 2296.73	0.004*
Lymp. count (103/mm3)	2448.00 ± 925.09	2850.87 ± 951.00	0.228
WBC count (103/mm3)	11406.00 ± 4549.25	8194.68 ± 2711.58	0.004*

*p < 0.05; Student t or Mann–Whitney U tests, SD: standard deviation; SAA: serum amyloid A, ESR: erythrocyte sedimentation rate; CRP: C-reactive protein, ANC: absolute neutrophil count; Lymp: lymphocyte; WBC: white blood cell.

## 4. Discussion

The study results showed that S100A12 protein level, ESR, and CRP level were significantly higher in children with FMF than in healthy controls. In addition, serum S100A12 levels were significantly higher in patients during the attack period, as were the WBC count, CRP level, ESR, SAA level, and fibrinogen level. Therefore, S100A12 level is a possible marker for monitoring inflammation in FMF patients. 

S100A12 is an important protein for evaluating the activity of several inflammatory diseases. This protein is distinctively increased in diseases such as systemic juvenile idiopathic arthritis and FMF, although it is also elevated in infection, malignancy, and some collagen diseases [9–14,16,17]. In a study of 56 children and adolescents with FMF, S100A12 levels had better indicative power than CRP level, ESR, and SAA level. The same study demonstrated that this protein’s level increased during the attack period in FMF patients but decreased with colchicine treatment and were higher in hereditary carriers without clinical findings than in the healthy population [18]. In another study, although S100A12 levels were higher in 29 adult FMF patients than in healthy controls, no difference was found in attack and attack-free FMF patients [19]. In our study, when we compared S100A12 levels of the attack-free, attack, and control groups, we found the highest S100A12 levels in the attack FMF group, followed by the attack-free FMF group and then the healthy controls. Although the acute-phase markers were within normal limits in our patients, they were higher than those in the control group. Colchicine treatment suppresses inflammation but is insufficient in some patients. This result indicated that S100A12 level, ESR, and CRP level can be used in the follow-up of such inflammation.

A comparison of attack versus attack-free patients revealed higher S100A12 level, WBC count, CRP level, fibrinogen level, and SAA level during FMF attack periods. A study showed that the use of colchicine decreases even extremely high S100A12 levels to near-normal levels after a week of treatment [18]. In addition, FMF attacks can last a few hours to 72 h, and the fact that the samples were not taken during the same stage as the patients’ attack periods may have contributed to this result.

Patients are generally asymptomatic except during attack periods. CRP and SAA levels are elevated during an attack, whereas ESR may not always increase [20]. However, subclinical chronic inflammation continues during the attack-free period [21–24]. In our study, comparison of attack-free patients with the healthy control group revealed that CRP, S100A12, and ESR levels were significantly higher in attack-free patients than in the control group. Although CRP levels, ESR, and SAA levels are within normal limits in FMF carriers, S100A12 levels are higher than normal healthy individuals and pick during attacks in patients with FMF [18,25,26]. Compared with traditional acute-phase reagents, S100A12 levels were higher in the silent phase of the disease [18]. These finding show that S100A12 can be used, like ESR and CRP, to follow-up patients with FMF and to diagnose FMF carriers. Taylan et al. [19] showed that S100A12, CRP, and SAA levels were correlated with each other in attack free period, in acute attack period of FMF and control patients. Accordingly, correlation analyses were performed on S100A12 and other acute-phase reactants in our study. When all patients were considered, a positive correlation was found between the S100A12 levels and CRP, ESR, and fibrinogen levels. No relationship was found between S100A12 levels and acute-phase reactants in the attack group. On the other hand, a positive correlation was found between S100A12 levels and ESR in the attack-free group. Although these findings were still within normal limits, especially in patients with FMF during the attack-free period, inflammation that can be demonstrated by ESR with S100A12 may continue.

ROC analysis evaluating S100A12 levels performed in a study that differentiated juvenile idiopathic arthritis patients with Kawasaki disease revealed an ROC curve of 0.97 (95% confidence interval, 0.89–1) [27]. In our study, when ROC analyses for patients with FMF were evaluated, S100A12 together with CRP, ESR, SAA, WBC, and fibrinogen are significant in the diagnosis and follow-up of FMF. As a result, SAA is the best predictor among acute-phase reactants. If the patient’s SAA value is 8.0, the patient may be diagnosed with FMF with 85.7% sensitivity, 93% specificity, and an 82.5% probability. Similarly, if the patient’s S100A12 value is 367, the diagnosis of FMF can be made with 64.3% sensitivity, 72.1% specificity, and 62% probability. Similar definitions can be made for CRP, ESR, fibrinogen, and WBC. According to these results, S100A12 is not superior to other acute-phase reactants, but similar to them, it can be used for diagnosis and monitoring.

Normal serum S100A12 levels are reportedly less than 120 ng/mL in healthy individuals [9,18]. However, some individuals in our control group had above-normal levels. S100A12 levels are known to increase in cases of infection, malignancy, and vasculitis [10,16,17]. The control group showed no evidence of disease. Recent studies have shown that S100A12 levels are high in healthy carriers of FMF [18,28]. The prevalence of FMF in our country is reportedly 1%, while 20% of its population carries the disease [3,4]. According to these results, the rate of FMF carriers may be higher among different geographical regions. In addition, the presence of FMF disease in the child was initially questioned through the medical history provided by the patient and his family, and no genetic analysis for FMF carrier status was performed in the control group; therefore, healthy controls with high S100A12 levels could have been asymptomatic FMF carriers. 

The most common mutation in FMF, M694V, is considered a risk factor for amyloid formation [2–4,6,17,18,29–31]. The MEFV gene and S100A12 activity are expressed as increased levels of interleukin-1 cytokines [7,8]. Interestingly, levels of this protein remain within normal limits in Muckle–Wells and neonatal onset multisystem inflammatory disease syndromes, which, like FMF, exhibit inflammation through interleukin-1 activity [32]. Other mechanisms that might increase S100A12 protein levels in FMF should be considered. In some studies of patients with FMF, S100A12 levels were the highest in patients with M694V homozygous or heterozygous mutations [18,19]. R202Q was the most commonly seen polymorphism in Turkey [31]. In our study, no significant relationship was found in the M694V mutation analysis, and this mutation was accompanied by the R202Q variation at a rate of 74.2%. One reason for this may be our small sample size, or that the dosage of colchicine therapy may differ widely in children, which has varying effects on S100A12 levels. Another reason could be the lack of research regarding the relationship between R202Q variations and S100A12 protein levels. 

In a study conducted by Stoler et al. [33] of 19 adult FMF patients (four were homozygous M694V mutations), S100A12 levels were high in those with homozygous M694V mutations. Similar results were obtained in another study [26]. In our study, WBC count, ESR, fibrinogen level, and S100A12 level were higher in M694V homozygous patients than in patients with other mutations. These results indicate that the M694V homozygous group is at higher risk for chronic inflammation and amyloidosis. According to these results, the persistence of chronic inflammation and the risk of amyloidosis are higher in M694V homozygous patients than the other patients. We can say that an M694V homozygous status carries a higher risk for chronic inflammation. In addition, the higher absolute neutrophil count, WBC count, S100A12 level, ESR, and fibrinogen level in the M694V homozygous group indicates that these patients are at higher risk for chronic inflammation. 

Limitations of this study: The study sample was limited in the number of patients, 

especially those during the FMF attack period. In addition, S100A12 levels were not measured in the same patients before and after the attack period. Our patient group received colchicine therapy, which is known to decrease S100A12 levels. Serum samples obtained before starting colchicine therapy could yield more accurate results, while certain associations could be revealed through mutation analyses. 

In conclusion, here we concluded that S100A12 levels are higher in FMF patients than in the healthy population and are higher during the acute attack than during the attack-free period. For this reason, measuring S100A12 levels may be helpful for diagnosing FMF and monitoring its attacks and may be an important marker for monitoring chronic inflammation in patients with FMF. We found no relationship between M694V mutation and serum S100A12 levels, but S100A12 levels were higher in homozygous M694V mutation patients with FMF than the patients with other mutations. Further studies including serum S100A12 samples obtained from a larger number of patients before colchicine therapy or samples taken from patients who received colchicine for the same duration could yield more accurate results regarding such correlations.

## Funding

No funding was received for this study. ELISA kits were purchased by the authors and analyzed in a private laboratory.

## Informed consent

The study protocol was approved by University of Health Sciences, Okmeydanı Training and Medical Research Hospital in accordance with the Declaration of Helsinki with ethical approval reference no: 22.01.2019/1107. Before becoming involved in the study, all participants who agreed to be involved in the study were given a cover letter describing the study objectives as well as a written informed consent form.
